# Editorial: Molecular mechanisms and treatment of monogenic skeletal disorders

**DOI:** 10.3389/fendo.2025.1666528

**Published:** 2025-08-07

**Authors:** Chao Xu, Weibo Xia, Peter Ebeling, Xiangbing Wang

**Affiliations:** ^1^ Department of Endocrinology, Shandong Provincial Hospital Affiliated to Shandong First Medical University, Jinan, China; ^2^ Department of Endocrinology, Key Laboratory of Endocrinology of National Health Commission, Peking Union Medical College Hospital, Chinese Academy of Medical Sciences & Peking Union Medical College, Beijing, China; ^3^ Department of Medicine, School of Clinical Sciences, Faculty of Medicine, Nursing & Health Sciences, Monash University, Clayton, VIC, Australia; ^4^ Divisions of Endocrinology, Metabolism, and Nutrition, Rutgers, The State University of New Jersey, New Brunswick, NJ, United States

**Keywords:** monogenic skeletal disorders, molecular mechanisms, treatment, bone research, gene mutation

Monogenic skeletal disorders are rare conditions caused by mutations in key genes involved in bone formation and mineralization, leading to a range of severe skeletal abnormalities such as bone deformities, fractures, and growth retardation (1). With numerous causative genes and highly variable symptoms, these disorders present considerable difficulties in clinical diagnosis and management. Advances in genetic sequencing technologies have greatly enhanced our ability to identify the underlying genetic mutations in these disorders, offering valuable insights for accurate diagnosis, genetic counseling, and personalized treatment strategies. This Research Topic highlights recent research efforts that improve our understanding of the genetic and therapeutic landscape of monogenic skeletal disorders.

An illustrative example is familial isolated hypoparathyroidism (FIH), caused by a bi-allelic mutation in the parathyroid hormone (PTH) gene (2). Mukhtar et al. describe a family where multiple members presented with elevated levels of biologically inactive PTH, leading to chronic hypocalcemia. This condition, unlike other forms of pseudohypoparathyroidism, lacked features of Albright hereditary osteodystrophy. Genetic testing revealed a c.128G>A mutation in the PTH gene, which was confirmed in other affected family members. Treatment with recombinant human PTH (teriparatide) successfully normalized calcium levels and significantly improved the patient’s symptoms and quality of life. This case underscores that high PTH levels in congenital hypocalcemia are not always indicative of receptor or post-receptor defects but can also result from biologically inactive mutated PTH, highlighting the essential role of genetic testing in diagnosing the underlying cause and guiding treatment.

Beyond clearly pathogenic mutations, some variants pose diagnostic uncertainty. Genetic sequencing has linked mutations to diseases, but not all genetic mutations result in clear clinical outcomes. Wu et al. examined whether mutations in the *ENPP1* gene are associated with bone mineralization and ectopic calcification. The *ENPP1* mutation (c.1352A>G, p.Y451C) was identified in a 74-year-old female patient, who was heterozygous for the mutation, with diffuse idiopathic skeletal hyperostosis (DISH) (3). The patient presented with ossification of the paraspinal ligaments, multiple vertebral fractures, and mild ectopic calcification in the Achilles tendons (3). However, Wu et al. found that one carrier of the Y451C mutation did not exhibit any clinical symptoms, despite both individuals being heterozygous carriers. Further experiments revealed that the mutation caused a significant reduction in enzymatic activity, yet the knock-in mouse model (*Enpp1^Y433C^*) did not exhibit significant skeletal abnormalities. These findings suggest that the pathogenic role of this variant remains uncertain, warranting further genotype-phenotype correlation studies.

As our understanding of genetic causes deepens, it opens the door to targeted treatments that can significantly improve patient outcomes. For example, burosumab, a monoclonal antibody against FGF23, has shown promise in treating FGF23 related mosaic disorders (4–6), where the overproduction of FGF23 leads to phosphate wasting and disrupted bone metabolism. Barbato et al. reviewed burosumab’s use in FGF23 related mosaic bone disorders and documented a case of an 11-year-old MAS patient with polyostotic fibrous dysplasia who received this therapy. The results indicated that burosumab provided beneficial effects in normalizing phosphate levels and improving bone mineralization in patients, offering a new therapeutic option for these disorders.

Similarly, Denosumab, a monoclonal antibody against receptor activator of NF-κB ligand (RANKL) has shown efficacy in improving bone mineral density (BMD) and reducing fracture incidence in osteogenesis imperfecta (OI) (7). Bisphosphonates have been the mainstay for increasing bone density in OI, but denosumab offers a more direct and reversible mode of action on osteoclasts. In Mei et al.’s study, denosumab significantly increased lumbar spine and femoral neck BMD after 12 months of treatment in a cohort of pediatric patients. However, careful risk-benefit assessments and age-specific dosing strategies are needed to optimize treatment outcomes, as rebound hypercalcemia can occur when the drug’s effects wear off.

Furthermore, gene therapy has emerged as a promising approach for treating genetic disorders by directly correcting the underlying genetic defects (8, 9). Autosomal recessive osteopetrosis (ARO) is a lethal infantile bone disorder often caused by mutations in *TCIRG1*, a gene essential for osteoclast function (10). The only current cure has been an allogeneic hematopoietic stem cell transplant (HSCT), which carries considerable risks and often only partially rescues the bone phenotype (11). In a study by Penna et al., lentiviral vector gene therapy, combined with non-genotoxic conditioning, was successfully used to correct the osteopetrotic bone phenotype in a mouse model of osteopetrosis. The researchers demonstrated that LV_PGK.TCIRG1-transduced oc/oc cells restored osteoclast function, allowing for long-term survival of transplanted mice and improving the bone phenotype while reducing extramedullary hematopoiesis.

Clinical studies have also provided valuable insights into the manifestations, diagnosis, and management of these disorders, ultimately improving patient outcomes. X-linked hypophosphatemia (XLH), a condition caused by mutations in the PHEX gene, typically presents in childhood with rickets, bone pain, short stature, and leg deformities like bowing (12). While medical therapy (phosphate supplements or burosumab) can improve the metabolic bone issues, many patients still require orthopedic interventions for limb deformities. Stauffer et al. used MRI and CT imaging to identify torsional pathologies that contribute to gait abnormalities and functional impairments in XLH patients. Notably, severe femoral retroversion was observed in a 2-year-old child before walking, suggesting that femoral torsional deformities may occur independently of mechanical load from walking. These findings underscore the importance of early evaluation and intervention in XLH patients to address these structural challenges.

In conclusion, the research presented in this Research Topic deepens our understanding of monogenic skeletal disorders, particularly in genetics and therapies. Ongoing advancements in genetic testing, molecular pathway elucidation, and innovative therapeutic approaches are laying the foundation for more effective and personalized treatments. Future research should focus on further clarifying the pathogenic mechanisms of gene mutations, evaluating the long-term effects of different treatment options, and exploring new therapeutic strategies. These efforts will provide more precise and effective treatment options for patients, ultimately improving their prognosis and quality of life.

## References

[1] Hannan FM Newey PJ Whyte MP Thakker RV. Genetics of skeletal disorders. In: Stern PH, editor. Bone Regulators and Osteoporosis Therapy. Springer International Publishing, Cham (2020). p. 325–51. doi: 10.1007/164_2020_350, PMID: 32166388

[2] Newey PJ Hannan FM Wilson A Thakker RV. Genetics of monogenic disorders of calcium and bone metabolism. Clin Endocrinol (Oxf). (2022) 97:483–501. doi: 10.1111/cen.14644, PMID: 34935164 PMC7614875

[3] Kato H Ansh AJ Lester ER Kinoshita Y Hidaka N Hoshino Y. Identification of ENPP1 haploinsufficiency in patients with diffuse idiopathic skeletal hyperostosis and early-onset osteoporosis. J Bone Miner Res Off J Am Soc Bone Miner Res. (2022) 37:1125–35. doi: 10.1002/jbmr.4550, PMID: 35340077 PMC9177665

[4] Apperley LJ Senniappan S. Burosumab therapy in a paediatric patient with McCune-Albright syndrome: A case report. Horm Res Paediatr. (2023) 96:341–8. doi: 10.1159/000527129, PMID: 36279852

[5] Gladding A Szymczuk V Auble BA Boyce AM. Burosumab treatment for fibrous dysplasia. Bone. (2021) 150:116004. doi: 10.1016/j.bone.2021.116004, PMID: 33984553 PMC8272883

[6] Sawamura K Hamajima T Kitoh H. Improvement of fibrous dysplasia after burosumab therapy in a pediatric patient with McCune-Albright syndrome: A case report. JBJS Case Connect. (2024) 14. doi: 10.2106/JBJS.CC.24.00279, PMID: 39303050

[7] Liu J Lin X Sun L Zhang Q Jiang Y Wang O. Safety and efficacy of denosumab in children with osteogenesis imperfecta—the first prospective comparative study. J Clin Endocrinol Metab. (2024) 109:1827–36. doi: 10.1210/clinem/dgad732, PMID: 38198649 PMC11180505

[8] Mingozzi F High KA. Therapeutic *in vivo* gene transfer for genetic disease using AAV: progress and challenges. Nat Rev Genet. (2011) 12:341–55. doi: 10.1038/nrg2988, PMID: 21499295

[9] Sayed N Allawadhi P Khurana A Singh V Navik U Pasumarthi SK. Gene therapy: Comprehensive overview and therapeutic applications. Life Sci. (2022) 294:120375. doi: 10.1016/j.lfs.2022.120375, PMID: 35123997

[10] Penna S Villa A Capo V. Autosomal recessive osteopetrosis: mechanisms and treatments. Dis Model Mech. (2021) 14:dmm048940. doi: 10.1242/dmm.048940, PMID: 33970241 PMC8188884

[11] Schulz A Moshous D. Hematopoietic stem cell transplantation, a curative approach in infantile osteopetrosis. Bone. (2023) 167:116634. doi: 10.1016/j.bone.2022.116634, PMID: 36470372

[12] Trombetti A Al-Daghri N Brandi ML Cannata-Andía JB Cavalier E Chandran M. Interdisciplinary management of FGF23-related phosphate wasting syndromes: a Consensus Statement on the evaluation, diagnosis and care of patients with X-linked hypophosphataemia. Nat Rev Endocrinol. (2022) 18:366–84. doi: 10.1038/s41574-022-00662-x, PMID: 35484227

